# Macrophage dysregulation in inflammatory bowel disease: cellular heterogeneity, pathogenic mechanism, and treatment

**DOI:** 10.3389/fimmu.2026.1837255

**Published:** 2026-05-21

**Authors:** Yuanyuan Yang, Haiying Zhu

**Affiliations:** Department of Cell Biology, Naval Medical University (Second Military Medical University), Shanghai, China

**Keywords:** cellular heterogeneity, inflammation, inflammatory bowel disease, intestinal macrophages, macrophage reprogramming

## Abstract

Inflammatory bowel disease (IBD) is sustained not only by dysregulated adaptive immunity, but also by profound disruption of the intestinal macrophage compartment. In healthy mucosa, macrophages maintain immune tolerance, support epithelial integrity, and coordinate repair. In IBD, however, these cells are reprogrammed into heterogeneous pathogenic states shaped by excessive monocyte recruitment, failed acquisition of resident programs, transcriptional and epitranscriptomic rewiring, metabolic stress, and persistent microenvironmental instruction. Macrophage behavior is continuously tuned by microbiota-derived metabolites, dietary signals, and reciprocal crosstalk with epithelial, stromal, and immune cells, placing macrophages at the center of a dynamic inflammatory circuit rather than at the end of a linear effector pathway. This review summarizes how macrophage dysregulation drives core pathological features of IBD, including cytokine amplification, defective microbial handling, epithelial barrier breakdown, fibrosis, and impaired mucosal healing. We further outline the current macrophage-directed therapeutic strategies, including targeted nanomedicine, metabolic reprogramming, biomimetic delivery systems, and vesicle- or gene-based approaches. Although these strategies remain constrained by lesion heterogeneity, incomplete human validation, and translational uncertainty, a more precise understanding of macrophage state architecture may provide a foundation for mechanism-based stratification and precision therapy in IBD.

## 1 Introduction

Inflammatory bowel disease, including Crohn’s disease and ulcerative colitis, is a group of chronic immune-mediated disorders characterized by persistent mucosal inflammation rather than intermittent symptomatic flares (1, 2). Its pathogenesis involves a complex interplay of genetic susceptibility, immune dysregulation, epithelial remodeling, oxidative stress, and disturbances in the microbiota and virome, which together sustain tissue injury and impair mucosal healing (3–5). Over time, this unresolved inflammation drives fibrosis, recurrent disease activity, and progressive complications, highlighting the need for mechanism-based therapeutic strategies in IBD management (6–8).

Within this framework, intestinal macrophages are central to disease initiation, persistence, and failed repair. Under homeostatic conditions, they act as sentinel phagocytes that integrate microbial and tissue-derived cues to maintain mucosal balance; however, disruption of their regulatory programs converts them into potent amplifiers of inflammation (9, 10). Disease chronicity is reinforced by defective monocyte-to-macrophage differentiation, sustained ETS2-associated inflammatory circuitry, and maladaptive polarization states, which together promote cytokine production, epithelial injury, and leukocyte recruitment (11, 12). Meanwhile, pathogenic macrophage states impair barrier restitution and uncouple resolution from tissue repair, while hybrid inflammatory-fibrotic phenotypes may further accelerate chronic remodeling (13–15).Intestinal macrophages represent a key target for mechanism-driven therapeutic intervention in IBD.

## 2 Cellular heterogeneity of intestinal macrophages in inflammatory bowel disease

### 2.1 Disease-associated macrophage states in the inflamed intestinal mucosa

In the inflamed intestinal mucosa, macrophage heterogeneity is not an M1/M2 continuum but a state space defined by recruitment, localization, and effector wiring. Single-cell and spatial profiling showed that the greatest divergence in IBD lies within myeloid cells, with macrophage subsets occupying discrete niches and engaging inflammatory fibroblast circuits (16). In UC, resident macrophages undergo a disappearance reaction under oxidative stress because SOD2 is scarce, whereas inflammatory macrophages replace them and redirect TNF production through networks with T and B cells (17). Inflamed UC mucosa is enriched for S100A8^+^ and IL1β^+^ macrophages linked to mucosal injury, while SPP1 macrophages align with CHI3L1^+^ fibroblasts at inflamed sites and track severity (18, 19). By contrast, M2d macrophages retain IL10 signaling, CCL3-CCR1 communication, and epithelial-healing potential (20). In active Crohn’s disease, IFNγ-polarized macrophages dominate, wound-healing IL-4^-^, IL-10^-^, and IL-13^-^programmed states contract, and TREM-1^+^ immature macrophages mark a pathogenic compartment (21, 22). CRIg^+^ macrophage loss permits microbial DNA-containing vesicles to enter mucosa and activate cGAS/STING signaling, whereas IL1β^+^ macrophages drive fibrosis through AREG-PI16^+^ fibroblast crosstalk, and tofacitinib non-response reflects IL-10-dependent macrophage hyperactivation (23–25).

### 2.2 Monocyte recruitment and defective differentiation into resident macrophage programs

In active IBD, circulating monocytes are recruited excessively to the intestinal mucosa yet fail to complete the CSF1R-dependent adaptation program required for differentiation into hyporesponsive resident macrophages, thereby favoring inflammatory amplification over mucosal tolerance (11, 26). Recent evidence suggests that multiple susceptibility loci converge on defective monocyte-to-macrophage education, with dysregulation of ETS2, NOD2-related pathways, and CSF1 signaling impairing acquisition of the anergic resident phenotype necessary for intestinal homeostasis (11). In Crohn’s disease, monocyte-derived cells also display aberrant adhesion and differentiation programs; notably, circFNDC3B is upregulated in peripheral monocytes and inflamed mucosa, where it modulates FNDC3B/TGF-β signaling and reshapes M2-like activation, indicating that defective differentiation is encoded at both transcriptional and post-transcriptional levels (27).

### 2.3 Transcriptional and genetic regulators shaping pathogenic macrophage phenotypes

Pathogenic macrophage phenotypes in inflammatory bowel disease do not arise as passive consequences of inflammation alone; rather, they are actively imposed by an interconnected regulatory architecture integrating inherited susceptibility, transcription factor networks, epitranscriptomic control, metabolic rewiring, and microenvironmental sensing. Human genetic studies support this model by demonstrating substantial inter-individual variation in macrophage transcriptional responses, with gene-specific defects in monocyte-derived macrophage adaptation to the intestinal milieu linked to IBD susceptibility, particularly through HLA-associated variation and differential inducibility of inflammatory mediators such as IL1B, IL23A, CXCL8, and NLRP3 (28). Among chromatin-associated regulators, SP140 appears particularly important, as its loss of function in Crohn’s disease derepresses topoisomerase activity, disrupts macrophage lineage fidelity, impairs antibacterial programs, and promotes intestinal pathology, thereby directly linking epigenetic reader dysfunction to pathogenic macrophage reprogramming (29). Epitranscriptomic mechanisms add a further layer of control. In gut macrophages, deficiency of the m6A reader YTHDC1 aggravates colitis by enhancing inflammatory responses while weakening epithelial barrier-supportive functions through regulation of RHOH and NME1 (30). Similarly, enterotoxigenic Bacteroides fragilis toxin suppresses FOXD3-dependent METTL3 transcription, reduces m6A modification, stabilizes ITGA5 expression, and thereby promotes inflammatory macrophage activation in IBD (31). Complementary studies indicate that distinct m6A regulatory modules may either exacerbate or restrain macrophage pathogenicity depending on their downstream targets: METTL3/YTHDF1-dependent m6A modification of SerpinB5 promotes M1 polarization and worsens ulcerative colitis through an FBXO32-dependent NF-κB pathway, whereas METTL14-mediated stabilization of TSC1 limits M1 skewing and supports a more reparative phenotype (32, 33).

Classical transcription factors remain central effectors within this broader regulatory network. ELF4 appears to exert protective effects by activating IL1RN transcription, suppressing inflammatory Th17 responses, and promoting M2 polarization, thereby constraining mucosal injury (34). Consistent with this, STAT6 signaling supports anti-inflammatory macrophage programming and preserves barrier function in experimental colitis, suggesting that disruption of this axis may contribute to the persistence of pathogenic macrophage states (35). Other regulators operate through immunometabolic and organelle homeostasis pathways. SAMHD1 deficiency promotes inflammatory macrophage polarization by disrupting autophagy-lysosomal homeostasis through the mTOR–MITF–CTSD axis, whereas FATS deficiency stabilizes HIF-1α, increases glycolytic enzyme expression, and drives M1 polarization, thereby linking defective intracellular stress adaptation to sustained inflammatory output (36). Mechanosensory and kinase-mediated signaling pathways provide additional regulatory inputs: Piezo1 activation reinforces NLRP3/NF-κB signaling and cytokine production, while Syk promotes reactive oxygen species generation, bactericidal dysfunction, and M1 polarization in colitis-associated macrophages (37, 38). Further layers of regulation include WTAP-mediated repression of Pannexin-1, which influences polarization and ferroptosis, and mesenteric adipose-derived exosomal miR-26b-3p, which targets TRIM33 to activate p38-MAPK signaling and intensify M1 polarization in Crohn’s disease-related inflammation (39, 40).These findings indicate that pathogenic intestinal macrophages in IBD emerge through convergent defects in transcriptional specification, epigenetic restraint, RNA modification, and signal-responsive metabolic circuitry, thereby defining a dense and potentially druggable regulatory network.

## 3 Pathogenic functions of macrophages in mucosal injury and disease progression

### 3.1 Pro-inflammatory cytokine and chemokine networks amplifying intestinal inflammation

In inflammatory bowel disease, macrophages exacerbate mucosal injury by establishing a self-reinforcing cytokine and chemokine network centered on IL-1β, TNF-α, IL-6, IL-18, and CXCL1, thereby sustaining leukocyte recruitment, epithelial damage, and chronic tissue inflammation (41–43). Within this network, IL-1β appears to function as a critical nodal mediator. In inflammatory macrophages during colitis, its production is enhanced through glycogen metabolism-dependent UDPG-P2Y14-STAT1 signaling, while concomitant inflammasome activation promotes caspase-1 cleavage and release of mature IL-1β, directly linking metabolic rewiring to escalating inflammatory output (41). This circuit is further reinforced by NLRP3 inflammasome activation, which not only augments IL-1β production but also stabilizes pathogenic macrophage polarization and broader intestinal immune disequilibrium (44, 45). Additional amplification arises from upstream signaling pathways. Activation of macrophage TLR4/NF-κB signaling drives increased secretion of TNF-α and IL-1β, whereas ETS2 promotes ulcerative colitis progression by intensifying this M1-associated inflammatory program (46, 47). Similarly, WNT2B-enriched macrophages activate NF-κB through competitive interaction with IKIP and IKKβ, further enhancing downstream inflammatory cytokine expression and aggravating intestinal inflammation (48). Beyond sterile inflammatory stimuli, microbial and viral signals provide further reinforcement. Desulfovibrio vulgaris flagellin activates the NAIP/NLRC4 inflammasome and induces macrophage pyroptosis, thereby worsening colitis and expanding pro-inflammatory signaling within the mucosa (49). Likewise, Epstein–Barr virus infection aggravates ulcerative colitis by promoting glycolysis-dependent macrophage pyroptosis with increased expression of NLRP3, IL-1β, and IL-18, thus linking infection-associated stress to cytokine storm-like amplification in the diseased intestine (43). Taken together, these findings indicate that macrophages in IBD do not merely secrete isolated inflammatory mediators; rather, they orchestrate an integrated cytokine–chemokine–inflammasome network that perpetuates mucosal injury and constitutes a central therapeutic target for interrupting disease progression.

### 3.2 Impaired microbial handling and epithelial barrier disruption

In IBD, intestinal macrophages not only amplify inflammatory signaling but also exhibit defective microbial handling, thereby perpetuating epithelial injury and barrier dysfunction. In Crohn’s disease, adherent-invasive Escherichia coli (AIEC) can survive and replicate within macrophages by adapting to phagolysosomal stress, forming intracellular bacterial communities and exploiting heterogeneous host-cell responses. As a consequence, macrophages are converted from bactericidal sentinels into intracellular reservoirs that sustain mucosal inflammation (50–52). This failure of microbial clearance is accompanied by oxidative and metabolic dysfunction, including ROS-driven M1 polarization and impaired antioxidant defense, both of which intensify inflammatory signaling and secondarily destabilize epithelial integrity (53, 54). Barrier injury is further propagated through macrophage-derived paracrine mechanisms. Exosomal miR-223 released from activated macrophages directly impairs epithelial barrier function by suppressing TMIGD1, while inflammatory macrophage programs promote tight-junction loss, increased epithelial permeability, and bacterial translocation in coculture and *in vivo* colitis models (55–57). Importantly, epithelial cells can further amplify this pathogenic loop by releasing ferritin-containing extracellular vesicles that are internalized by macrophages through MSR1, thereby inducing oxidative stress and inflammatory activation that worsen colitis severity (58). Conversely, restoration of macrophage homeostasis improves both microbial control and barrier maintenance. Butyrate enhances AIEC phagocytosis while suppressing macrophage inflammatory outputs through HDAC3 inhibition, and SCFA-producing bacterial consortia similarly reinforce mucosal barrier function by skewing macrophages toward reparative phenotypes (59, 60). These findings indicate that impaired microbial handling and epithelial barrier disruption are not parallel abnormalities, but mechanistically coupled consequences of macrophage dysregulation in IBD.

### 3.3 Macrophage contributions to fibrosis, tissue remodeling, and defective mucosal healing

Macrophages drive fibrostenotic progression in IBD not only by sustaining inflammation but also by directly instructing matrix-producing and reparative compartments. In Crohn’s disease, intestinal E. coli-derived yersiniabactin depletes macrophage zinc, stabilizes HIF-1α, and induces profibrotic transcriptional programs in macrophages that localize to fibrotic strictures, linking microbial metal sequestration to tissue remodeling (61). In parallel, osteopontin-activated MerTK^+^ macrophages amplify ERK/TGF-β1 signaling, promote fibroblast activation, and are enriched in inflamed and stenotic bowel, while MerTK inhibition attenuates fibrosis *in vivo (*62). Recent work further identifies IL1β^+^ macrophages as a stromal-activating subset that secretes AREG to activate PI16+ fibroblasts, and TGF-β1 can also drive macrophage-myofibroblast transition with α-SMA and collagen production, creating a self-reinforcing fibrotic niche (24, 63). Beyond fibrosis, dysfunctional macrophages also impair healing: LPS-primed macrophages induce ACTA2 and COL1A1 expression and collagen accumulation in intestinal organoid cocultures (64), whereas M2 macrophage ferroptosis restricts anti-inflammatory activity and delays mucosal repair in ulcerative colitis (65). Conversely, reparative metabolic reprogramming of macrophages restores epithelial regeneration and mucosal healing (66, 67).

## 4 Microenvironmental control of macrophage reprogramming in inflammatory bowel disease

### 4.1 Microbiota-derived signals and metabolic cues driving macrophage dysfunction

In IBD, microbiota-derived metabolites and diet-shaped metabolic signals actively reprogram macrophages rather than merely mirroring inflammation. In Crohn’s disease, Achromobacter pulmonis identified in creeping fat induces macrophage IDO1 expression and enriches L-kynurenine, and macrophage-sourced kynurenine then acts through adipocyte AHR to drive mesenteric adipogenesis, showing that bacterial translocation can redirect macrophage tryptophan metabolism toward a pathogenic extraintestinal niche (68). In ulcerative colitis, protective microbial circuits run in the opposite direction: Lactobacillus acidophilus generates ursodeoxycholic acid, which inhibits M1 macrophage polarization through RapGap/PI3K-AKT/NF-κB signaling (69), whereas Radix Aucklandiae reshapes the microbiota by enriching Lactobacillus reuteri and increasing indole-3-aldehyde, which binds AHR, suppresses TNF-α, IL-6 and IL-1β, and restrains M1 activation (70). Additional metabolites reinforce this anti-inflammatory rewiring: agmatine, a microbiota-derived polyamine, lowers LPS-driven cytokine and NO production, reduces M1 cells, increases M2 cells, and partly acts through histone deacetylase inhibition (71); probiotic Lactobacillus plantarum-derived extracellular vesicles similarly promote M2 polarization, increase IL-10 and TGF-β, suppress histamine, IL-6 and TNF-α, and are accompanied by remodeling of lysine degradation with increased oxoadipic acid (72); Akkermansia muciniphila drives M1-to-M2 conversion through the SCFAs-SLC52A2/FFAR2 pathway while reducing oxidative stress, apoptosis, pyroptosis and necroptosis (73). By contrast, pathogenic metabolic pressure pushes macrophages toward inflammatory injury: 7-ketositosterol from ultra-processed foods blocks ALKBH5 nuclear translocation, downregulates GCLM, impairs glutathione synthesis, and triggers macrophage ferroptosis with accumulation of MDA, Fe2+ and ROS, thereby aggravating colitis severity (74). At the intracellular level, HMGB1 further consolidates this dysfunctional state by repressing Cpt1a, increasing glycolysis, reducing fatty acid oxidation and favoring M1 polarization (75), whereas restoration of metabolic checkpoints by FFAR1/FFAR4 agonism, resveratrol-mediated arginine rewiring, or sodium butyrate-induced SIRT1/GPX4/SLC7A11 signaling shifts macrophages back toward lipid-oxidative, ferroptosis-resistant and inflammation-resolving programs (76–78).

### 4.2 Crosstalk between macrophages and epithelial, stromal, and immune compartments

Within the inflamed intestinal mucosa, macrophage reprogramming is governed not by macrophage-intrinsic programs alone, but by sustained reciprocal signaling with epithelial, stromal, and immune compartments. Epithelial cells are active participants in this process. For example, microbiota-remodeling interventions that enhance IL-10RA signaling in macrophages strengthen macrophage–epithelial communication, suppress NF-κB activation, and improve tight-junction integrity, indicating that epithelial barrier restoration and macrophage polarization are mechanistically coupled in ulcerative colitis (79). In parallel, epithelial-derived apoptotic cargo can also shape macrophage behavior, as LXRα activation promotes macrophage efferocytosis of injured intestinal epithelial cells while simultaneously restraining inflammatory polarization (80).

**Figure 1:**
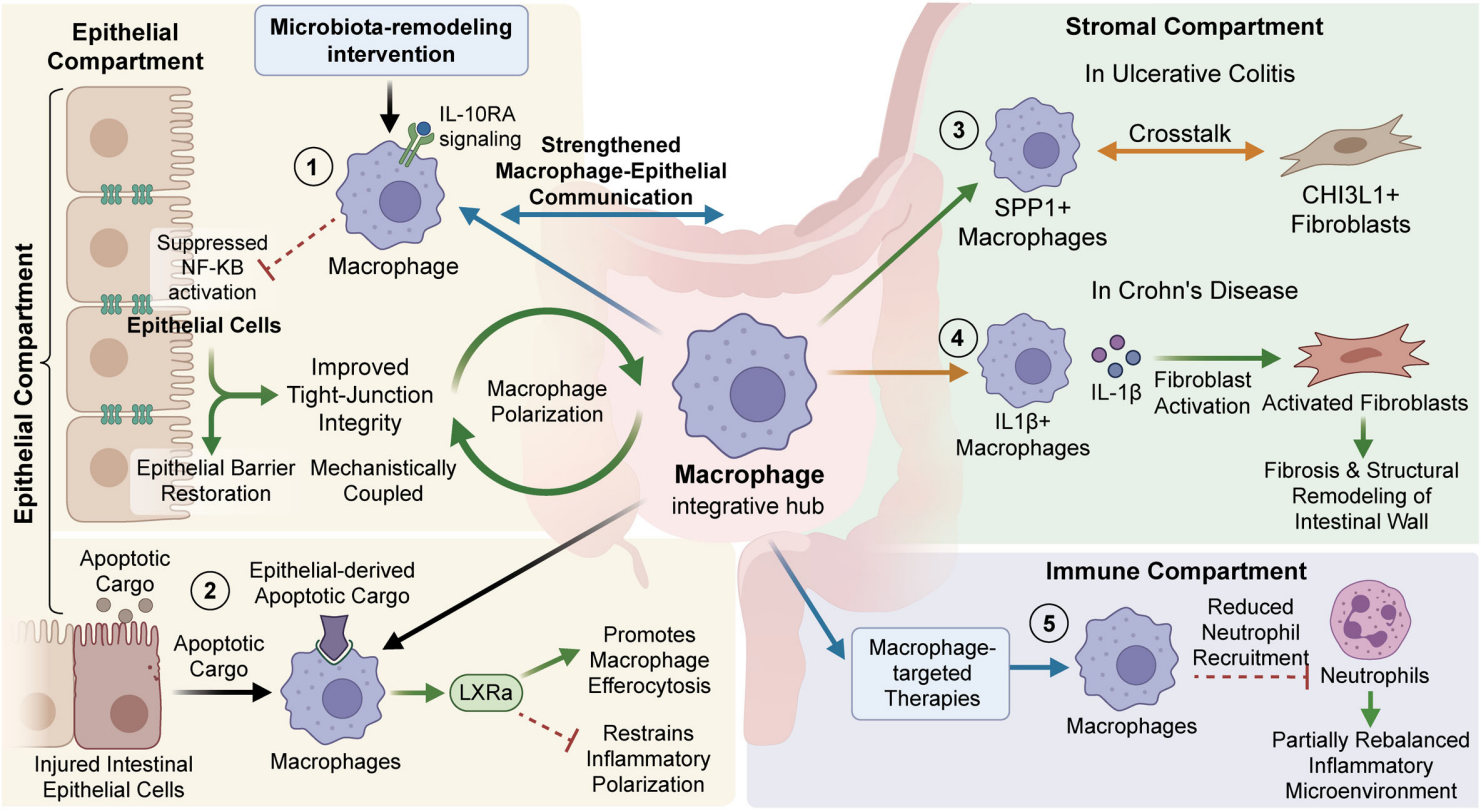
Roles of macrophages in inflammatory bowel disease.This schematic describes the bidirectional signaling between macrophages and intestinal compartments. In the epithelial compartment, microbiota-remodeling/LXRα activation influences barriers and inflammation. In stromal, macrophage subtypes drive UC/CD specific fibroblast responses and fibrosis. Finally, targeted therapies reduce neutrophil recruitment in the immune compartment to favor repair.

Stromal interactions further consolidate pathogenic macrophage states and extend their consequences into tissue remodeling. In ulcerative colitis, SPP1^+^ macrophages exhibit enriched crosstalk with CHI3L1^+^ fibroblasts, whereas in Crohn’s disease, IL1β^+^ macrophages drive fibroblast activation, thereby linking immune dysregulation to fibrosis and structural remodeling of the intestinal wall (19, 24). These local multicellular circuits are integrated into a broader immune network, as macrophage-targeted therapies can reduce neutrophil recruitment and partially rebalance the wider inflammatory microenvironment (81).These observations position macrophages as central integrative hubs within the IBD microenvironment, where continued bidirectional communication with epithelial, stromal, and immune cells reinforces either pathogenic inflammation or, when appropriately redirected, mucosal repair (**Figure 1**).

## 5 Therapeutic targeting of intestinal macrophages

### 5.1 Current macrophage-centered therapeutic strategies

Current macrophage-centered therapeutic strategies in inflammatory bowel disease increasingly extend beyond nonspecific immunosuppression toward selective reprogramming, depletion, or metabolic correction of pathogenic intestinal macrophages. A major theme across recent studies is the effort to shift inflammatory M1-like macrophages toward reparative M2-like states while simultaneously improving drug retention at inflamed mucosal sites and minimizing systemic toxicity (82). This principle is exemplified by mucoadhesive mesalamine prodrug nanoassemblies that exploit cathepsin β-rich inflammatory macrophages for local drug release, thereby enhancing intestinal retention, suppressing inflammatory signaling, and promoting M2 polarization in colitis models (83). Similarly, calcium-carbonate mineralized liposomes co-delivering a ferroptosis inhibitor were designed to couple M2 induction with ferroptosis restraint, thereby addressing the vulnerability of anti-inflammatory macrophages to oxidative death and improving the M2/M1 ratio in experimental IBD (84). Other delivery systems employ colon-targeted or pH-responsive formulations to enhance macrophage-directed efficacy, including chitosan-coated artesunate, which suppresses TLR4/NF-κB signaling while engaging STAT6-dependent M2 polarization and barrier protection, and berberine-loaded macrophage membrane-derived nanovesicles that prolong colonic retention, attenuate oxidative stress, and modulate macrophage phenotype (35, 85). Taken together, these studies indicate that macrophage targeting is being developed not merely as a means of anti-inflammatory drug delivery, but as a strategy to reshape the inflammatory niche through cell-selective uptake, intracellular signaling control, and restoration of epithelial integrity (86).

A second emerging direction involves targeting the metabolic and organelle programs that sustain pathogenic macrophage activation. Rather than conceptualizing macrophage polarization as a fixed binary state, recent work frames inflammatory macrophages as metabolically rewired cells whose glycolysis, oxidative stress, ferroptosis susceptibility, and mitochondrial dysfunction are therapeutically tractable (87). Bioinspired adhesive microparticles delivering γ-glutamylcysteine alleviate ulcerative colitis by suppressing macrophage ferroptosis, restraining M1 reprogramming, and restoring barrier function and microbial balance, thereby establishing ferroptosis as a relevant therapeutic node rather than a mere downstream consequence of inflammation (88). In parallel, polysaccharide-iron nanozymes redirect macrophage glucose metabolism from glycolysis toward oxidative phosphorylation through PI3K/Akt modulation, reducing NF-κB-driven inflammatory outputs while also addressing iron deficiency anemia as a common IBD comorbidity (89). Mitochondria-targeted delivery platforms further refine this strategy by delivering quercetin into the mitochondria of intestinal M1 macrophages through cascade targeting, thereby attenuating inflammatory cascades while promoting barrier restoration and microbiota re-equilibration (86). Gene- and vesicle-based approaches add yet another mechanistic layer: laminarin-mediated oral delivery of miRNA-223 promotes macrophage repolarization through dectin-1-directed uptake, whereas human umbilical cord mesenchymal stem cell-derived exosomes inhibit macrophage inflammasome activity by activating the SIRT1-FXR axis and reducing FXR acetylation (82, 90). These findings suggest that effective macrophage-directed therapy in IBD may require simultaneous correction of immunometabolism, oxidative injury, and intracellular stress responses rather than simple suppression of cytokine production.

A further frontier involves biomimetic and combinatorial systems that integrate macrophage targeting with barrier repair, microbiota remodeling, or interruption of intercellular inflammatory circuits. Macrophage-mimetic nanoplatforms have been developed to restore both the intestinal epithelial barrier and the gut vascular barrier, thereby reducing bacterial translocation and interrupting feed-forward inflammatory amplification that conventional epithelial-centered therapies may fail to control adequately (91). Prebiotic-engineered oral nanoplatforms similarly combine selective uptake by folate receptor-overexpressing M1 macrophages with microbiota remodeling and photodynamic therapy, highlighting the therapeutic potential of simultaneously eliminating inflammatory macrophages and reshaping the luminal ecosystem that sustains their activation (92). Other biomimetic formulations, such as macrophage-mimetic glycyrrhizic acid-functionalized liposomes for celastrol delivery, have shown encouraging performance in active targeting, immune evasion, and biosafety, while also reducing pro-inflammatory cytokine production and supporting barrier repair (91). Beyond nanomedicine, some natural compounds likewise seem to modulate macrophage-driven pathology through more defined mechanisms. Dendrobium officinale polysaccharide has been linked to the SENP1-SIRT3 axis, whereas atractylenolide III appears to act through direct targeting of IL-17RA; in preclinical models, both attenuated M1 polarization and relieved mucosal injury (92). Even so, current support for these approaches still rests largely on *in vitro* and animal data. Key obstacles to clinical translation include scalable manufacturing, consistent macrophage selectivity in heterogeneous human lesions, long-term safety, and the unresolved question of whether results from ulcerative colitis can be generalized to Crohn’s disease. For that reason, the field is likely to move not toward a universal macrophage-targeted therapy, but toward more carefully stratified interventions built around dominant macrophage programs in particular disease contexts, including inflammasome activation, ferroptosis, glycolytic rewiring, and failed reparative polarization (**Supplementary Table 1**).

## 6 Conclusion

Macrophage dysregulation is a core organizing mechanism in inflammatory bowel disease, linking persistent inflammation, defective resolution, barrier failure, and maladaptive tissue remodeling. Rather than representing a uniform population, intestinal macrophages in IBD occupy dynamic and heterogeneous states shaped by impaired monocyte differentiation, transcriptional and metabolic rewiring, microbiota-derived cues, and sustained intercellular crosstalk within the mucosal niche. These alterations drive cytokine amplification, defective microbial handling, fibrosis, and impaired healing. Emerging macrophage-centered therapies therefore hold considerable promise, but meaningful clinical translation will depend on mechanistic stratification, human tissue validation, and interventions tailored to dominant pathogenic macrophage programs. A more resolved macrophage framework may provide a rational foundation for next-generation precision therapy in IBD.
